# Pipeline Terracotta Microbial Fuel Cell: Organic Content Biosensor and Energy Harvesting Device Integrated in Wastewater Pipeline

**DOI:** 10.3390/bios14050224

**Published:** 2024-04-30

**Authors:** Trang Nakamoto, Dung Nakamoto, Kozo Taguchi

**Affiliations:** Department of Electrical and Electronic Engineering, Ritsumeikan University, Kusatsu 525-8577, Japan; n-trang@fc.ritsumei.ac.jp (T.N.); ayma.aime@gmail.com (D.N.)

**Keywords:** MFC, MFC sensor, integrated, clay, Co-MnO_2_

## Abstract

Wastewater pipelines are present everywhere in urban areas. Wastewater is a preferable fuel for renewable electricity generation from microbial fuel cells. Here, we created an integrated microbial fuel cell pipeline (MFCP) that could be connected to wastewater pipelines and work as an organic content biosensor and energy harvesting device at domestic waste-treatment plants. The MFCP used a pipeline-like terracotta-based membrane, which provided structural support for the MFCP. In addition, the anode and cathode were attached to the inside and outside of the terracotta membrane, respectively. Co−MnO_2_ was used as a catalyst to improve the performance of the MFCP cathode. The experimental data showed a good linear relationship between wastewater chemical oxygen demand (COD) concentration and the MFCP output voltage in a COD range of 200–1900 mg/L. This result implies the potential of using the MFCP as a sensor to detect the organic content of the wastewater inside the wastewater pipeline. Furthermore, the MFCP can be used as a long-lasting sustainable energy harvester with a maximum power density of 400 mW/m^2^ harvested from 1900 mg/L COD wastewater at 25 °C.

## 1. Introduction

The burgeoning global population, coupled with the rapid pace of urbanization, has led to the production of vast quantities of domestic wastewater. This phenomenon directly results from the higher density of inhabitants in urban areas and the corresponding increase in domestic activities that contribute to wastewater generation. The relentless march of industrialization further exacerbates the situation, which not only contributes to wastewater volumes but also intensifies the demand for energy [1]. As we look to the future, the question of energy supply becomes increasingly pressing. Traditional methods of energy generation, predominantly reliant on the combustion of fossil fuels, are now under intense scrutiny. The reason for this is twofold: firstly, the finite nature of fossil fuel reserves poses a challenge for sustainable long-term energy provision, and secondly, and perhaps more critically, the combustion process releases substantial quantities of carbon dioxide (CO_2_) gas into the atmosphere. The accumulation of CO_2_ and other greenhouse gases is a significant contributing factor to the global warming crisis, an issue that has far-reaching implications for the stability of our climate and the health of our planet [2,3]. In light of these concerns, there has been a discernible shift in focus towards renewable energy sources. The adoption of renewable energy is seen as a pivotal strategy in mitigating the effects of climate change. By harnessing energy from sources such as the sun, wind, and water, we can significantly reduce our reliance on fossil fuels and, consequently, our carbon footprint. Renewable energy offers a pathway to a more sustainable and environmentally friendly energy landscape that aligns with the urgent need to address the challenges posed by climate change [3,4]. 

Moreover, wastewater treatment is another issue caused by the increasing population. The treatment requires a significant amount of energy to process [5,6]. Therefore, if we can harvest energy from wastewater, it will be a sustainable solution for this issue. Consequently, a microbial fuel cell (MFC) has emerged as a promising technology because it uses living microorganisms in the wastewater to convert organic matter available in the wastewater into electrical energy. The working principle of MFCs is based on redox reactions in the anode and the cathode. Bacteria catalyze the oxidation reactions of organic matter in the anode chamber, producing electrons and protons. The anode collects the electrons from these reactions, and due to the electromotive force, the electrons flow to the cathode through an external circuit connected between the two electrodes to create an electric current. At the cathode, oxygen in the air reduces the electrons with the help of cathodic catalysts [7]. 

MFCs have attracted considerable attention as a green technology for renewable energy generation, wastewater treatment, biosensing, and bioremediation [5,8,9]. The main advantages of MFCs is their ability to operate under mild conditions, such as ambient temperature and pressure, and their ability to utilize a wide range of organic substrates, including various types of wastewaters [10,11,12]. Wastewater is a ubiquitous and abundant source of organic matter that can be used as a fuel for MFCs. However, wastewater also poses some challenges for MFCs, such as the presence of toxic compounds and the variability of composition and concentration [13]. 

Usually, a pipeline system transports wastewater to treatment plants, where wastewater is treated, and clean water is discharged to the environment after treatment. During the treatment process, the water quality of the wastewater needs to be tested regularly through indexes such as biological oxygen demand (BOD) or chemical oxygen demand (COD) [14]. BOD stands for the dissolved oxygen that microbes need to break down the organic matter in water, and standard BOD analysis requires 5 days to complete. COD stands for oxygen used when the water sample is chemically oxidized. BOD and COD are usually directly related to each other. For example, domestic wastewater’s COD/BOD ratio is typically between 1.5 and 2.0 [15]. However, the current methods for measuring BOD or COD cannot monitor the water online at the source [16]. This calls for the development of low-cost sensor devices that can provide real-time data on the BOD or COD of water [17], especially a device that can connect directly to the wastewater pipeline and sense the organic content of the water inside the pipeline. Such a device would help reduce the cost of regular monitoring of the water quality at wastewater treatment plants [6]. 

In this paper, we propose a novel concept of integrating MFCs into wastewater pipelines, creating a microbial fuel cell pipeline (MFCP) that can serve as both a biosensor and an energy harvester. The MFCP consists of a terracotta membrane that is shaped like a pipeline and has an anode and a cathode attached to its inner and outer surfaces, respectively. The wastewater flows inside the pipeline and acts as the anolyte, while the ambient air serves as the catholyte. The MFCP can sense the organic content of the wastewater, represented by the chemical oxygen demand (COD) concentration, by measuring the output voltage signal. The MFCP can also generate electricity from wastewater and power other low-power devices, such as remote sensor networks and Internet of Things (IoT) devices. The MFCP is a simple, low-cost, and scalable system that can be easily installed and connected to existing wastewater pipelines. The MFCP offers a potential solution for simultaneous wastewater monitoring and energy recovery.

## 2. Materials and Methods

### 2.1. Pipeline Terracotta Membrane Fabrication 

**Figure 1:**
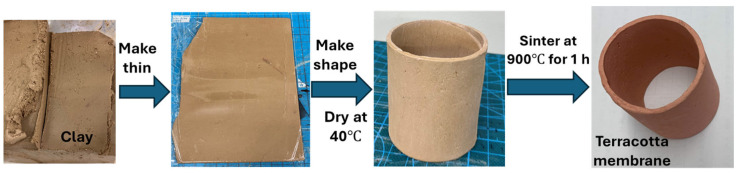
Fabrication process of the terracotta pipeline membrane.

In this research, we meticulously crafted a terracotta membrane (also known as a separator) using typical clay sourced from Bijutsu Shup-pan Educational Co., Ltd. in Tokyo, Japan. The entire fabrication process, as illustrated in Figure 1, involved several crucial steps. Initially, the raw clay material was carefully flattened into a thin layer, achieving a remarkable thickness of only 2 mm. The resulting clay sheet was shaped to resemble a pipeline, emphasizing its practical applicability in various engineering contexts. 

To enhance its structural integrity and thermal stability, the clay membrane underwent a controlled drying process at a moderate temperature of 40 °C for a duration of 15 to 20 h. Subsequently, the dried clay was subjected to a high-temperature sintering treatment at 900 °C for precisely 1 h. This transformative step converted the clay into a robust and durable terracotta separator, ready to withstand the rigors of real-world applications.

Interestingly, despite its solid nature, the terracotta separator exhibited a unique property: it allowed water to permeate through its microstructure. This intriguing behavior makes it an ideal candidate for serving as an effective cation exchange membrane for MFCs. By regulating water transport, the terracotta separator ensures proton exchange, facilitating efficient electrochemical reactions at the cathode–anode interface. As a result, our terracotta-based separator contributes to the overall performance and longevity of MFCs.

### 2.2. Cathodic Catalyst Synthesis and Characterization

In this research, we employed cobalt–manganese dioxide (Co−MnO_2_) supported on carbon black powder (Co−MnO_2_/C) as an economical and efficient catalyst for the air cathode of the MFCP. The utilization of Co−MnO_2_/C aims to enhance the overall performance and cost-effectiveness of the MFCP. The synthesis of the Co−MnO_2_/C catalyst was carried out using a straightforward redox method, which can be summarized as follows: Two separate solutions were meticulously prepared in deionized water: one containing 50 mL of 0.1 M potassium permanganate (KMnO_4_, Wako Pure Chemical Industries, Ltd., Tokyo, Japan) and the other with 50 mL of 0.1 M cobalt nitrate hexahydrate (Co(NO_3_)_2_ 6H_2_O, Wako Pure Chemical Industries, Ltd., Japan). These solutions were gradually mixed dropwise under slow magnetic stirring at a controlled temperature of 70 °C for a duration of 1 h. This step facilitated the redox reaction between the Co and Mn ions, leading to the formation of the Co−MnO_2_/C catalyst. Subsequently, 0.5 g of carbon black was added to the mixture, followed by an additional 10 min of stirring to ensure uniform dispersion. The resulting solution was allowed to sediment, leading to the precipitation of the Co−MnO_2_/C catalyst, which was carefully separated and isolated. Finally, the freshly synthesized Co−MnO_2_/C catalyst underwent controlled drying at 60 °C for 20 h to remove residual moisture. The photo image of the dried Co−MnO_2_/C powder is visually depicted in Figure S1 of the Supporting Information, providing valuable insights into its morphology and particle size distribution.

Cyclic voltammetry (CV) was used to evaluate the effect of the catalyst on oxygen reduction reaction (ORR) performance. CV was measured using a potassium ferricyanide electrolyte with a Ag/AgCl reference electrode, a Pt counter electrode, and a carbon sheet working electrode coated with and without the Co−MnO_2_/C catalyst. The voltage scan was performed forwardly at a scan rate of 50 mV/s and a potential range of −1 V to 1 V.

Moreover, a scanning electron microscope (SEM) was used to observe the surface morphology of the Co−MnO_2_/C catalyst powder. It was also compared to the surface morphology of carbon black powder without Co-MnO_2_ coating.

### 2.3. MFCP Configuration and Fabrication

**Figure 2:**
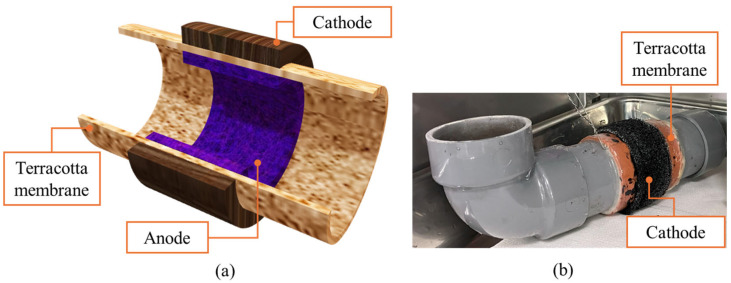
The configuration of an MFCP (**a**) and a photo image of an MFCP attached to a pipeline (**b**).

Let us delve into the structure of the MFCP, as depicted in Figure 2a. This innovative energy conversion system comprises several essential components: a terracotta membrane, an anode, and a cathode. The heart of the MFCP lies in its pipeline-shaped terracotta membrane. This specialized material serves as a crucial separator, effectively segregating the anode and cathode compartments. The terracotta membrane ensures selective ion transport while preventing direct contact between the anode and the cathode. The anode was positioned on the inside of the terracotta membrane. It serves as the site where the microbial oxidation of organic matter occurs. The anode facilitates the release of electrons, which contribute to the overall electricity generation process. The cathode was attached to the outside of the terracotta membrane. Here, oxygen reduction reactions take place, completing the electrochemical circuit and enabling the MFCP to generate power. In summary, the MFCP’s elegant design ensures efficient ion transport, microbial activity, and electricity production—all within the confines of its terracotta membrane. 

The electrodes of the MFCP were fabricated based on low-cost carbon-based materials [18,19]. To fabricate the anode, a specialized conducting paste was applied onto a 5 mm thick urethane foam substrate, which was subsequently dried. The conducting paste formulation involved a precise mixture of 2 g of carbon fiber and 2 g of coconut-based activated carbon powder combined with 10 mL of CNT ink (N7006L from KJ Specialty Paper, Ltd., Tokyo, Japan). The ink was used as a conductive binder. Carbon fiber and activated carbon powder help enhance the conductivity and surface area of the anode, respectively. Similarly, the cathodic material was prepared by blending 1 g of Co-MnO_2_/C catalyst with 10 mL of the same CNT ink. This cathodic mixture was then coated onto another 5 mm thick urethane foam substrate and dried. The resulting anode and cathode had surface areas of 40 cm^2^ and 50 cm^2^, respectively. To ensure efficient current collection, stainless steel wire was employed as the anodic and cathodic current collectors.

Following the preparation of the anode and cathode components on the terracotta membrane, the next crucial step involved their integration into a fully functional module. As depicted in Figure 2b, the anode–cathode assembly was securely attached to a pipeline structure, forming a cohesive energy conversion unit. This innovative system holds immense promise for sustainable energy applications and environmental monitoring. Notably, an alternative module shape is visually presented in Figure S2 in the Supporting Information, highlighting the versatility and adaptability of the MFCP design. 

### 2.4. Experimental Methods 

BOD and COD of organic wastewater have a propositional relationship [15]. Due to the absence of a BOD measurement system, we opted to utilize COD as a representative parameter for assessing the organic content in the wastewater. The determination of COD followed the standard protocol: digesting reagent vials (specifically, Hach TNT822) and conducting the reaction in a Hach DRB200 reactor. Subsequently, the COD levels were quantified using a Hach DR3900 spectrophotometer. This comprehensive approach ensures the accurate characterization of the organic load in the wastewater, contributing valuable insights to environmental monitoring strategy.

A lysogeny broth (LB) medium was used to simulate organic content typically found in domestic wastewater. The LB medium was prepared by mixing 10 g/L of tryptone, 5 g/L of yeast extract, and 10 g/L of NaCl in water. Subsequently, the entire mixture underwent rigorous sterilization at 120 °C for 1 h. Notably, the COD of this simulated wastewater can be precisely regulated by adjusting the concentration of the LB medium within the water matrix. This controlled manipulation allows us to mimic various organic load scenarios, providing valuable insights into wastewater treatment processes and environmental impact assessments.

The MFCP module underwent rigorous testing at the surrounding temperature range of 25–26 °C. To drive electricity generation, we harnessed the metabolic activity of a diverse bacterial consortium extracted from muddy soil, serving as the electrogenic bacteria source for the MFCP’s anode [8]. To evaluate its sensing capabilities, we systematically exposed the MFCP to varying COD levels, spanning from 200 mg/L to 1900 mg/L. Each specific COD value was maintained for a continuous 24 h measurement period. 

A 1 kΩ external resistor was connected between the two electrodes of the MFCP, and the voltage dropped on the resistor was monitored. In addition, the maximum power density of the MFCP was calculated based on Ohm’s Law using voltages measured at different external resistances and the surface area of the anode electrode, as explained in our previous paper [20]. Various external resistances of 10 kΩ, 5 kΩ, 2 kΩ, 1 kΩ, 0.7 kΩ, 0.5 kΩ, 0.25 kΩ, 0.1 kΩ, 0.08 kΩ, and 0.04 kΩ were used for this measurement. Moreover, the results are averaged for presentation in this paper, and the differences between the three separate measurements are shown as error bars in graphs.

## 3. Results

### 3.1. Co-MnO_2_/C Cathodic Catalyst

**Figure 3:**
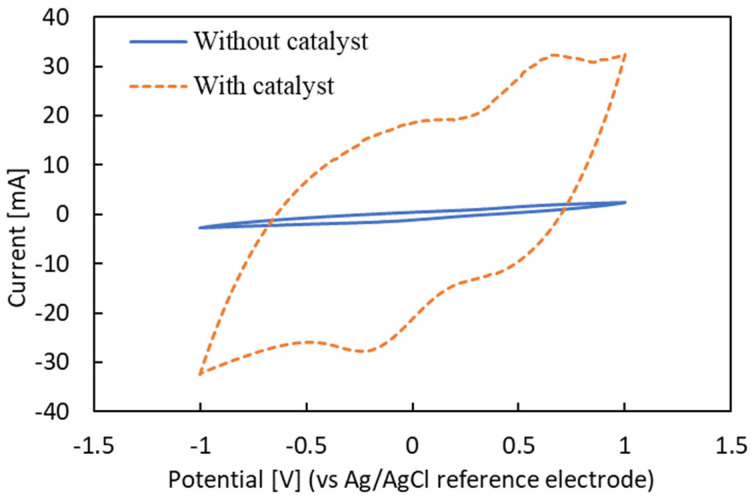
CV curves of the cathode material with and without the Co−MnO_2_/C cathodic catalyst. The measurement was conducted at a scan rate of 50 mV/s.

Figure 3 shows CV curves generated by carbon sheets with and without loading the Co-MnO_2_/C catalyst. It can be seen that the current generated by the carbon sheet with the catalyst was much higher than that of the carbon sheet without the catalyst. The ORR peak of the working electrode with the catalyst was at the potential of −0.23 V and the current of −27.75 mA, which is 17.3 times higher than the current generated by the working electrode without the catalyst (−1.60 mA). This result confirmed the effectiveness of the Co−MnO_2_/C catalyst on the ORR performance.

**Figure 4:**
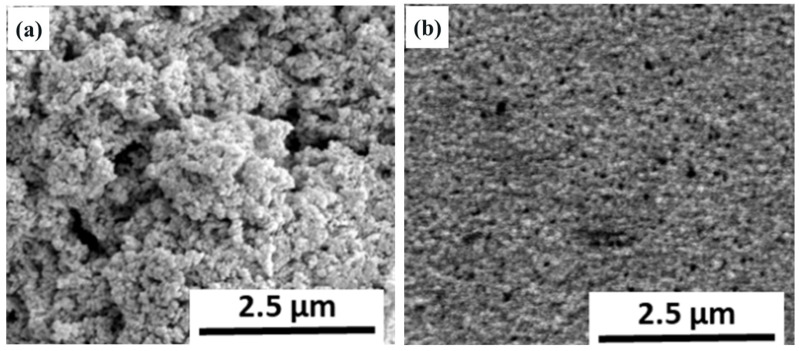
SEM images of (**a**) Co−MnO_2_/C catalyst compared with (**b**) carbon black.

Upon examining the SEM images of the catalyst surface compared with that of carbon black, as depicted in Figure 4, a notable difference was observed. The Co−MnO_2_/C catalyst exhibited a significantly more porous structure. This porosity is indicative of an enhanced surface area, which can be attributed to the presence of the Co−MnO_2_ catalyst. The observed increase in porosity suggests that the catalyst plays a crucial role in augmenting the surface area of the electrode. The underlying mechanism likely involves the intercalation of cobalt ions within the layers of manganese oxide (MnO_2_), which promotes the development of a more complex three-dimensional framework. This structural evolution is not merely superficial; it has profound implications for the functionality of the cathode, particularly in terms of its ORR performance, as discussed above.

The expanded surface area resulting from the catalyst’s action provides a more significant number of active sites for the ORR process. This, in turn, facilitates a more efficient interaction between the reactants and the cathode, thereby potentially leading to an improvement in the overall electrochemical performance of the cathode. Such enhancements are critical for the advancement of the air cathode in MFCs, where the efficacy of the ORR process is a pivotal factor.

The Co−MnO_2_/C catalyst’s contribution to the cathode’s morphology is substantial, as evidenced by the SEM imagery. The catalyst’s ability to induce a more porous structure by the intercalation of cobalt ions results in a beneficial increase in surface area. This morphological enhancement resulted in an ORR performance improvement, marking a significant step forward in the optimization of catalytic materials for the air cathode in MFCs. 

### 3.2. Organic Content Sensing Capability

**Figure 5:**
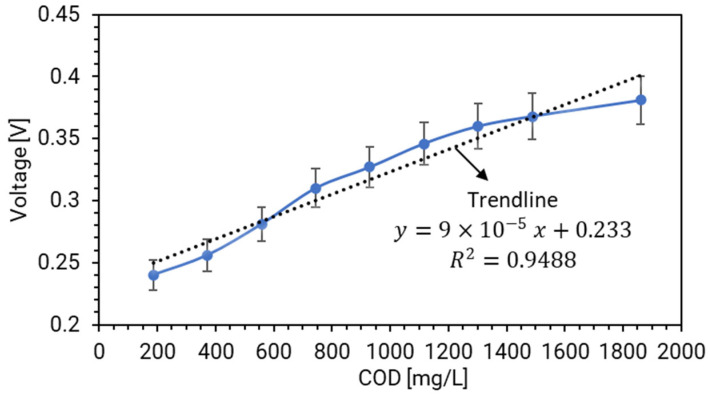
The direct relationship between the output voltage of the MFCP and the COD of the wastewater (error bars represent the voltage variation during the measurement period).

In the context of the MFCP, a detailed study was conducted to observe the relationship between the output voltage under a specific discharging condition and the fluctuations in the COD levels. The experimental setup involved the MFCP operating under a continuous discharging condition with a resistance of 1 kΩ. The findings of this experiment are shown in Figure 5. The experiment started with the MFCP handling wastewater with a COD concentration of 200 mg/L. At this initial stage, the MFCP was able to generate an output voltage of 240 mV. As the experiment progressed, the COD concentration within the wastewater pipeline was incrementally increased. Remarkably, when the COD level reached a peak of 1900 mg/L, the MFCP’s output voltage demonstrated a proportional increase, climbing to a notable 380 mV.

This pattern of voltage escalation in tandem with rising COD levels unveiled a compelling linear relationship, suggesting a direct correlation between the two variables. To substantiate this observation, a regression analysis was performed, which yielded an R^2^ value of 0.95. This high R^2^ value is indicative of a strong linear relationship, thereby reinforcing the initial hypothesis of a direct correlation between the output voltage and COD levels.

The practical implications of this discovery are significant. By monitoring the output voltage of the MFCP, one can deduce the COD concentration in the wastewater pipeline with a high degree of accuracy. This method presents a substantial advantage over traditional sampling and analysis techniques, which are often labor-intensive, time-consuming, and financially burdensome. The ability to predict COD levels through voltage readings simplifies the monitoring process, making it a more efficient and cost-effective approach for managing wastewater treatment systems.

In essence, the experiment has not only demonstrated a clear linear relationship between the MFCP’s output voltage and the COD concentration but has also highlighted a transformative method for assessing wastewater quality. This innovative approach aims to streamline operations and reduce operational costs in wastewater management facilities, marking a significant advancement in environmental monitoring techniques. 

### 3.3. Energy Harvesting from Wastewater

The power density generated by the MFCP was also measured to determine its renewable energy harvesting capacity. For this measurement, the COD of wastewater was set at 1900 mg/L, initiating the measurement from an open circuit voltage (OCV) condition. When the external resistance was changed from 10 kΩ to 40 Ω, the voltage of the MFCP dropped from 0.65 V to 0.25 V, and based on the voltage and resistance values, the current and power could be calculated using Ohm’s law. By dividing the calculated current and power by the surface area of the anode, the current density and power density of the MFCP could be obtained. 

**Figure 6:**
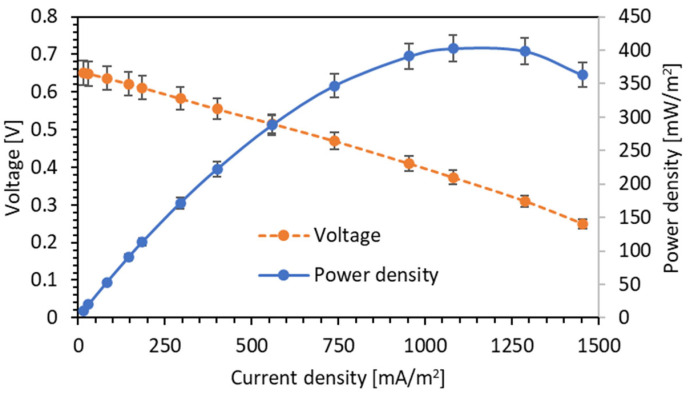
Polarization curve and power density curve of the MFCP module (error bars represent the deviation of the measured results).

Figure 6 shows the polarization and power density curves obtained from the MFCP. The MFCP demonstrated a remarkable performance, achieving a maximum power density of approximately 400 mW/m^2^. This significant achievement underscores the MFCP’s capacity to harness renewable energy effectively. The high power density indicates that the MFCP is a strong contender for applications in sustainable power generation. Its ability to convert bio-waste into electricity in an environmentally friendly manner makes it a promising power source for self-powered low-power devices, such as remote sensor networks and IoT devices.

Furthermore, the maximum power output of the MFCP was obtained when the external resistance was at 80 (Ω). According to the maximum power transfer theorem, the maximum power transfer occurs when the load resistance equals the source resistance. Therefore, it can be inferred that the internal resistance of the MFCP is also 80 Ω. This value of internal resistance is notably low, especially when compared to other reported microbial fuel cells (MFCs). Many MFCs reported in the literature often exhibit internal resistances that span a wide range, typically from hundreds to thousands of Ohms [21,22]. The relatively low internal resistance of the MFCP is a significant advantage, as it allows for a higher power output.

## 4. Discussion

From Figure 5, the amount of electricity generated by the MFCP correlated proportionally to the concentration of organic matter (represented through COD) inside the anode chamber. This intriguing correlation underscores the MFCP’s potential utility as a COD/BOD biosensor for online water quality monitoring applications [17,23]. Notably, the MFCP operates autonomously, devoid of any external power sources, and requires minimal maintenance. Its versatility extends to monitoring organic pollution levels across diverse water bodies in various aqueous environments. Unlike the biosensors developed in [24], which exhibited unstable output due to sludge accumulation on the cathodes, the MFCP circumvents this issue. The key lies in the MFCP’s unique design: the cathode remains insulated from direct contact with wastewater. Consequently, the MFCP demonstrates remarkable stability and resilience over extended periods. In fact, our rigorous testing spanning over 8 months has revealed no significant degradation in its operational performance. This longevity positions the MFCP as a promising solution for sustainable water quality assessment and environmental monitoring.

Although terracotta-based membranes have been used to make a floating BOD sensor [25], here, we focus on developing a simple fabrication process of the terracotta with controllable thickness and shape. To the best of our knowledge, this research is one of the first to develop a pipeline-pluggable MFC using a terracotta-based membrane. 

In this research, the experimental temperature was deliberately set at about 25 °C, which was lower than the bacteria’s favorable temperature of 30–40 °C [26]. This deliberate choice mirrors real-world scenarios, where ambient temperatures typically hover around 25 °C or even lower. It is worth noting that, at lower operating temperatures, the output of MFCs tends to decrease significantly due to reduced bacterial metabolic activity [26]. Therefore, at higher operating temperatures, it can be expected that the MFCP can harvest more power density than the result obtained in Figure 6.

Moreover, there are some limitations of this study that we want to address in future research. First, the limitation of detection (LOD) has not been precisely determined. In the present experimental setup, the lowest COD of 200 mg/L was used, and the output voltage of the MFCP module was at 0.24 V level (1 kΩ continuous discharging voltage). Therefore, it can be predicted that a much lower COD level can be detected if we set the LOD of MFCP voltage at 0.1 V level. Second, while our study has successfully explored the linear relationship between COD and voltage within the range of 200–1900 mg/L, we acknowledge that this trend may deviate from linearity as COD approaches the upper limit of our testing range. In Figure 5, the linear trend tended to become a curve when COD increased near the maximum testing range. It can be expected that there will be a point where the relationship is no longer linear. Third, as the MFCP uses an air cathode, it can only be used in open-air environments, such as the outputs of domestic wastewater pipelines. The limited amount of accessible air will affect the MFCP output and, thus, the accuracy of the sensor. Finally, in the present study, the MFCP was tested solely in controlled laboratory conditions. The natural wastewater would be much more complex, and the ambient temperature would vary widely. Therefore, in future work, in situ experiments are needed to collect on-site data and evaluate the feasibility of the biosensor. 

Further research can be developed to integrate organic content indicator systems with the MFCP module, such as a visual LED indicator circuit [8] or a wireless signal transceiver circuit [27,28]. These indicator systems can harness the power harvested by the MFCP, eliminating the need for external power sources. These research directions hold immense promise for advancing sustainable energy technologies and environmental monitoring practices. 

## 5. Conclusions

This research proposed a MFCP fabricated in a pipeline shape based on a terracotta separator. The terracotta separator was fabricated using a simple method, and it provided structural support for the MFCP. The MFCP could be used as an online COD/BOD biosensor because it exhibited a linear relationship between the output voltage and the concentration of the organic load of wastewater. The MFCP could also be used as an energy harvester to harvest electrical energy from wastewater produced by electrogenic bacteria living inside wastewater. In the experiment, a maximum power density of 400 mW/m^2^ was harvested from 1900 mg/L COD wastewater by the MFCP working at 25 °C. The MFCP stands out as a promising candidate for sustainable power generation applications, paving the way for more research and development in this field. 

## Data Availability

Data supporting this study are included within the article and/or supporting materials.

## Supplementary Materials

The following supporting information can be downloaded at: https://www.mdpi.com/article/10.3390/bios14050224/s1, Figure S1: Photo image of the Co-MnO_2_/C catalyst after drying; Figure S2: Photo image of another shape of an MFCP module. The MFCP is placed in the middle of the module.
