# Supplementary material for: Pipeline Terracotta Microbial Fuel Cell: Organic Content Biosensor and Energy Harvesting Device Integrated in Wastewater Pipeline
Source: Biosensors (Basel). 2024 Apr 30;14(5):224. doi: 10.3390/bios14050224 (PMC11117956; doi:10.3390/bios14050224)
Supplement: Supplementary file 1 [file biosensors-14-00224-s001.zip › biosensors-2912971-supplementary.pdf]

Supporting Information

Pipeline Terracotta Microbial Fuel Cell: Organic Content Biosensor and Energy Harvesting Device Integrated in Wastewater Pipeline

Trang Nakamoto[1], Dung Nakamoto[1], and Kozo Taguchi[1,*]

[1] Department of Electrical and Electronic Engineering, Ritsumeikan University, Kusatsu, Shiga 525-8577, Japan; n-trang@fc.ritsumei.ac.jp

*Correspondence: taguchi@se.ritsumei.ac.jp; Tel.: (+81)77-561-5178

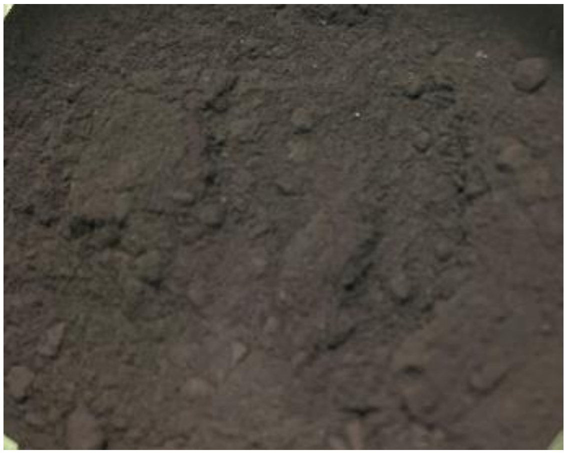

Figure S1. Photo image of the $Co\text{-}MnO_2/C$ catalyst after drying

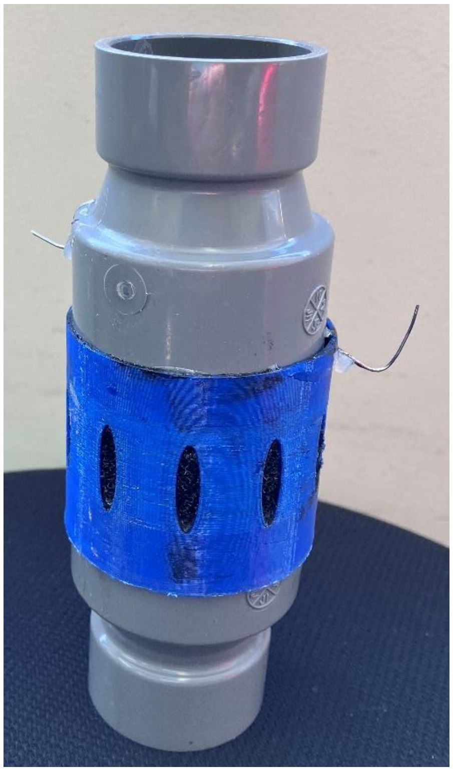

Figure S2. Photo image of another shape of an MFCP module. The MFCP is placed in the middle of the module.
